# Infrequent, low magnitude HIV-specific T cell responses in HIV-uninfected participants in the 1% tenofovir microbicide gel trial (CAPRISA004)

**DOI:** 10.1186/1742-4690-9-S2-P225

**Published:** 2012-09-13

**Authors:** WA Burgers, TL Muller, A Kiravu, V Naranbhai, S Sibeko, L Werner, Q Abdool Karim, SS Abdool Karim

**Affiliations:** 1 University of Cape Town, Cape Town, South Africa; 2 CAPRISA, Nelson R Mandela School of Medicine, Durban, South Africa

## Background

Macaque studies of antiretroviral-containing microbicide gels administered rectally or vaginally followed by SIV challenge have documented priming of SIV-specific T cell responses in the blood of protected animals. This concept has been termed “chemo-vaccination”, where aborted viral replication is thought to leave an immune footprint of exposure, which may augment protection provided by microbicides/PrEP. We investigated whether T cell responses were detectable in women participating in CAPRISA004 1% tenofovir microbicide trial, which showed 39% efficacy in reducing HIV acquisition.

## Methods

Thirty-eight HIV-uninfected participants were selected based on consistently high gel use and a high number of recorded sex acts over the duration of the trial. Cryopreserved PBMC were stimulated with HIV-1 peptide pools based on the HIV-1 clade C proteome, and IFN-gamma production was measured by the ELISPOT assay. Positive response were defined as >55 SFU/106 PBMC. Samples were tested at the visit at which preceding monthly coital activity was the participant’s highest, and at study exit. Assays were conducted blinded to placebo or tenofovir arm.

## Results

T cell responses were detected in 1/18 tenofovir and 2/13 placebo participants at the high gel use visit. Responses were of low magnitude (between 60 and 100 SFU/106 PBMC), and directed at peptide pools from HIV Gag, Pol, Nef and Env. T cell responses were not detected at the exit visit. These data suggest that HIV-specific responses are infrequently detected in blood in uninfected participants from a clinical trial of a vaginal microbicide, and where present, are of low magnitude and transient.

## Conclusion

Magnitude and timing of viral exposure may account for differences in detecting systemic T cell responses between preclinical studies in non-human primates and a human clinical trial of a vaginal microbicide.

